# The Effects of PP2A Disruption on ER-Mitochondria Contact and Mitochondrial Functions in Neuronal-like Cells

**DOI:** 10.3390/biomedicines11041011

**Published:** 2023-03-26

**Authors:** Phaewa Chaiwijit, Kwanchanok Uppakara, Nithi Asavapanumas, Witchuda Saengsawang

**Affiliations:** 1 Department of Physiology, Faculty of Science, Mahidol University, Bangkok 10400, Thailand; 2 Chakri Naruebodindra Medical Institute, Faculty of Medicine Ramathibodi Hospital, Mahidol University, Samut Prakan 10540, Thailand; 3 Department of Basic Biomedical Sciences, Dr. William M. Scholl College of Podiatric Medicine, Rosalind Franklin University of Medicine and Science, North Chicago, IL 60064, USA

**Keywords:** PP2A, ER-mitochondria contacts, MAMs, calcium, mitochondrial dynamics, Alzheimer’s disease, Tau

## Abstract

Mitochondria-associated membranes (MAMs) regulate several cellular processes, including calcium homeostasis and mitochondrial function, and dynamics. While MAMs are upregulated in Alzheimer’s disease (AD), the mechanisms underlying this increase remain unknown. A possible mechanism may include dysregulation of protein phosphatase 2A (PP2A), which is reduced in the AD brain. Furthermore, PP2A has been previously reported to modulate MAM formation in hepatocytes. However, it is unknown whether PP2A and MAMs are linked in neuronal cells. Here, to test the correlation between PP2A and MAMs, we inhibited the activity of PP2A to mimic its low levels in AD brains and observed MAM formation, function, and dynamics. MAMs were significantly increased after PP2A inhibition, which correlated with elevated mitochondrial Ca^2+^ influx and disrupted mitochondrial membrane potential and mitochondrial fission. This study highlights the essential role PP2A plays in regulating MAM formation and mitochondrial function and dynamics for the first time in neuronal-like cells.

## 1. Introduction

Alzheimer’s disease (AD) is the most widespread neurodegenerative disorder and is characterized by cognitive and behavioral impairment. Familial AD (FAD) is known to be caused by mutations in specific genes but represents less than 5% of AD cases. Conversely, the etiology of sporadic AD (SAD), which is more common, remains unclear. Several lines of evidence have demonstrated that mitochondrial dysfunction is an early pathological feature of both FAD and SAD prior to the formation of amyloid plaques and neurofibrillary tangles [1,2]. These findings illustrate that prevention or alleviation of mitochondrial dysfunction might be a promising target for therapeutic interventions in AD.

Mitochondria-associated membranes (MAMs) are subcellular compartments that enable close-proximity communication between the ER and mitochondria. MAMs regulate several fundamental cellular functions, including lipid exchange, Ca^2+^ transfer, and mitochondrial function and dynamics [3,4,5,6]. Emerging evidence shows that deregulated MAM formation is associated with numerous neurodegenerative diseases. Fibroblasts of AD patients with a presenilin mutation and presenilin knockout cell models of AD both exhibited increased MAMs and mitochondrial dysfunction [7,8]. In addition, findings in the brain of an amyloid precursor protein (APP)_Swe/Lon_ mouse model showed upregulated MAM-associated proteins before the appearance of plaques [9]. The upregulated MAMs induced sphingolipid turnover and changed the lipid composition of both MAMs and mitochondria, resulting in impaired mitochondrial respiratory activity. The main function of MAMs is calcium transfer during ER calcium release. This results in a 100-fold increase in mitochondrial Ca^2+^ levels without a noticeable increase in cytosolic calcium. Ca^2+^ is released from the ER through inositol 1,4,5-trisphosphate receptors (IP3Rs) and is taken up by mitochondria via voltage-dependent anion channels (VDACs). In fibroblasts from AD patients with a presenilin mutation, mitochondrial Ca^2+^ uptake was elevated due to an upregulation of MAMs [10]. This increase in mitochondrial Ca^2+^ has been shown to trigger oxidative stress and neurodegeneration [11]. The oxidative stress can promote mitochondrial damage, which might be the cause of the reduction in both mitochondrial activity and ATP production found in AD. Moreover, the expression levels of MAM proteins involved in calcium signaling were altered in post-mortem analyses of human AD brains and in AD mouse model brains [9]. Apart from calcium transfer, MAMs are involved with the regulation of mitochondrial dynamics, including fusion and fission [12,13,14], which are also altered in the brains of AD patients [15,16]. Since MAMs appear to play a role in several dysfunctional cellular processes in AD, dysregulation of MAMs might be a key pathological feature for therapeutic targeting. However, the underlying factors causing aberrant MAM functions in AD remain unknown.

Protein phosphatase 2A (PP2A) is a major serine/threonine phosphatase in the brain. A reduction in the expression and activity of PP2A has been found in the post-mortem brains of patients with AD [17,18,19]. Moreover, PP2A dysfunction has been linked to several pathological hallmarks of AD, including tau hyperphosphorylation, amyloidogenesis, and synaptic deficits [20,21]. Apart from AD, a study on hepatocytes showed that PP2A activity was activated during high-glucose exposure. This increase in PP2A activity reduced the number of MAMs and disrupted mitochondrial Ca^2+^ transfer, resulting in mitochondrial dysfunction [22]. This finding suggests that PP2A activity is linked to MAMs, which led us to investigate the effect of PP2A reduction on MAM formation in neuronal-like cells. Here, we reveal that okadaic acid-induced PP2A inhibition affects MAM formation and function. This evidence highlights another important pathological process that PP2A regulates in neuronal-like cells apart from tau hyperphosphorylation and accumulation. Understanding the relationship between PP2A and MAMs may provide more information on how MAMs are altered in AD brains.

## 2. Materials and Methods

### 2.1. Cell Culture

The SH-SY5Y human neuroblastoma cell lines were purchased from the American Types Culture Collection (ATCC, Manassas, VA, USA) and maintained in Dulbecco’s Modified Eagle Medium: Nutrient Mixture F-12 (DMEM-F12) supplemented with 10% Fetal bovine serum (FBS) and 1% antibiotic-antimycotic. Cells were cultured at 37 °C in a 5% CO_2_ incubator.

### 2.2. Immunoblotting

SH-SY5Y cells were plated in 6-well plates at a density of 800,000 cells/well in growth medium. After treatment with okadaic acid, cells were washed with cold PBS and harvested. Cells were then centrifuged at 3000× *g* at 4 °C for 5 min. The supernatant was removed, and the cell pellet was lysed using a modified RIPA buffer (50 mM Tris pH 8.0, 150 mM NaCl, 1 mM EDTA, 1% TritonX-100, and 0.1% SDS with freshly prepared protease inhibitor (mini-complete)) (Roche, Basel, Switzerland). An equal amount of protein sample in each well was separated by sodium dodecyl sulfate-polyacrylamide gel electrophoresis (SDS-PAGE) and transferred to Polyvinylidene difluoride (PVDF) membranes. The membranes were blocked from non-specific binding with 5% *w/v* non-fat dry milk in TBST 0.05% Tween. The membranes were then incubated with the following primary antibodies: anti-phospho Tau (AT8) (1:1000, Thermo Fisher Scientific, Waltham, MA, USA), anti-Tau (1:1000, Abcam, Cambridge, UK), anti-phospho DRP1, anti-DRP1, anti-mfn1, anti-mfn2, anti-opa1 (1:1000, Santa Cruz Biotechnology, Inc., Dallas, TX, USA), anti GSK3β, and anti pGSK3β (1:1000 and Cell Signaling, Danvers, MA, USA), at 4 °C overnight. The membranes were then washed and incubated with anti-rabbit or anti-mouse HRP-conjugated secondary antibodies (Jackson ImmunoResearch Laboratories Inc., West Grove, PA, USA) at room temperature for 1 h. Finally, protein signals were detected using Luminata crescendo Western HRP substrate, and the membranes were exposed to X-ray film. The signal intensities of individual samples were analyzed using ImageJ software. 

### 2.3. Proximity Ligation Assay (PLA)

Cells were cultured on Millicell EZ SLIDE 8-well glass (MilliporeSigma, Burlington, MA, USA) at a density of 35,000 cells/well in growth medium followed by okadaic acid treatment for 10 h and were then fixed with 4% paraformaldehyde for 20 min. Blocking was performed with a blocking solution acquired from the Duolink^®^ PLA assay kit (MilliporeSigma, Burlington, MA, USA) for 1 h at 37 °C. The cells were incubated with primary antibodies VDAC1 (1:600, Abcam, Cambridge, UK) and IP3R-1 (1:500, Abcam, Cambridge, UK) in antibody diluent overnight at 4 °C in a humidified chamber. Then, the cells were rinsed with Wash Buffer A for 10 min (2×). Next, PLUS and MINUS secondary PLA probes, both rabbit and mouse immunoglobulins diluted in antibody diluent, were added for 1 h at 37 °C. The incubation was followed by 5 min washes with two exchanges of Wash Buffer A again. After this, the slides were incubated with the Duolink^®^ ligation mix for 30 min at 37 °C and thereafter washed with Wash Buffer A two times for 5 min each. The Duolink^®^ amplification mix was applied to the slides for 100 min at 37 °C. Subsequently, the slides were washed twice for 10 min each time with Wash Buffer B. Later, the cells were incubated with Alexa Fluor™ 488 Phalloidin (1:400, Thermo Fisher Scientific, Waltham, MA, USA) in 1% BSA for 1 h at room temperature, followed by 5 min washes with PBS. Nuclei were stained with Duolink^®^ Mounting Media with DAPI, and stained cells were mounted on microscope slides, sealed, and imaged with a confocal laser scanning microscope. Quantification of PLA red fluorescent dots was performed using ImageJ software.

### 2.4. Ca^2+^ Measurements

SH-SY5Y cells were plated in 8-well imaging chambers at a density of 35,000 cells/well in growth medium for 48 h, followed by treatment with okadaic acid. Cells were incubated with 1 μM Rhod2-AM or 3 μM Cal-520 dye in an external solution (145 mM NaCl, 2 mM KCl, 5 mM NaHCO_3_, 1 mM MgCl_2_, 2.5 mM CaCl_2_, 10 mM glucose, and 10 mM Na-HEPES, pH 7.25) at 37 °C for 15 min and 1 h, respectively. Then, the cells were washed with the external solution for 5 min at 37 °C. Rhod2 and Cal-520 fluorescence signals were time-lapse imaged at 581 nm (Rhod2) and 488 nm (Cal-520) at 880 ms intervals at 37 °C using the Zeiss LSM 900 confocal laser scanning microscope (Carl Zeiss, Jena, Germany). To trigger Ca^2+^ release from ER stores through IP3 receptors (IP3Rs), 100 μM Oxotremorine-M (Oxo-M) was added to the imaging chamber for 2 min. The dynamics of mitochondrial Ca^2+^ levels were calculated as the relative differences in Rhod2 or Cal-520 fluorescence intensity compared to baseline fluorescence (pretreatment). The BD FACSCanto™ flow cytometer (BD Biosciences, Franklin Lakes, New Jersey, USA) was used to determine basal levels of mitochondrial Ca^2+^ in the experiment.

### 2.5. TMRM Assay

SH-SY5Y cells were plated in 6-well plates at a density of 800,000 cells/well, followed by treatment with 10 μM of okadaic acid. After trypsinization, the cells were incubated with 10 nM tetramethylrhodamine, methyl ester (TMRM) in the external solution at 37 °C for 45 min. To depolarize mitochondrial membrane potential, the cells were incubated with 10 μM carbonyl cyanide chlorophenylhydrazone (CCCP) for 15 min before TMRM staining. Then, the cells were washed with the external solution and centrifuged at 500× *g* for 5 min. Cell pellets were resuspended with PBS containing 0.5% BSA. TMRM-positive cells were detected with a BD FACSCanto™ flow cytometer. 

### 2.6. Statistical Analysis

Statistical analysis was calculated using GraphPad Prism version 8.0.0 (GraphPad Software, San Diego, CA, USA). All results are expressed as means ± SEMs. Student’s *t*-tests were performed for comparisons between two independent datasets. The criteria for statistical significance were established, where * *p* < 0.05, ** *p* < 0.01, and *** *p* < 0.001.

## 3. Results

### 3.1. PP2A Inhibition Increased MAM Formation

**Figure 1:**
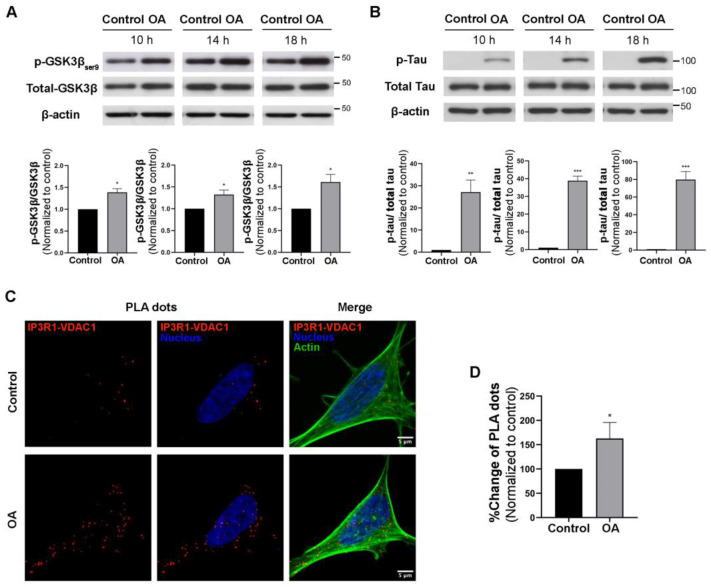
Effects of PP2A inhibition on pGSK3β, pTau, and MAMs in SH-SY5Y cells. Western blot analysis of (**A**) pGSK3β/total GSK3β expression and (**B**) pTau/total Tau expression in control cells and okadaic acid-treated cells at 10 h, 14 h, and 18 h. The bar graphs show quantifications of the blots normalized with b-actin. (**C**) Interactions between IP3R1 and VDAC1 (PLA dots) in okadaic acid-treated cells (10 h) compared to untreated cells. Representative images showing maximum-intensity projections of confocal image stacks. (**D**) Bar graph showing quantification of PLA signals in okadaic acid-treated cells compared to untreated cells. Data represent means ± SEMs of three independent experiments. * *p* < 0.05, ** *p* < 0.01, *** *p* < 0.001 (Student’s *t*-test). Scale bars are 5 μm.

To investigate the effects of PP2A activity modulation on MAM formation and function in neuronal cells, we inhibited PP2A in SH-SY5Y cells using okadaic acid. Okadaic acid is a PP2A inhibitor that has been widely used as a tool to study the underlying mechanism of AD pathologies [23]. To verify the PP2A inhibition effect of okadaic acid, we first determined the phosphorylation of glycogen synthase kinase-3β (GSK-3β) and tau, which have been shown to be increased by PP2A inhibition [24,25]. The cells were treated with 10 nM okadaic acid for 10, 14, and 18 h. Increases in GSK-3β phosphorylation at Ser9 and tau phosphorylation at Ser202 and Thr205 were observed after 10 h of PP2A inhibition, indicating that PP2A activity was inhibited (Figure 1A,B). To investigate whether MAMs are altered in the presence of okadaic acid, interactions between IP3Rs on the ER membrane and voltage-dependent anion channels (VDACs), well-known protein complexes at MAM contact sites on the outer mitochondrial membrane, were measured using a PLA. Red fluorescence dots represented IP3R1/VDAC1 interactions, as shown in Figure 1C. After 10 h of okadaic acid treatment, the IP3R1/VDAC1 interactions were significantly increased by up to 150% compared to the control (Figure 1D), indicating that PP2A inhibition increased the number of MAM contacts. The specificity of the PLA was verified using an IP3R1 primary antibody or VDAC1 primary antibody alone (Figure S1).

### 3.2. PP2A Inhibition Regulates Mitochondrial Calcium Transfer

**Figure 2:**
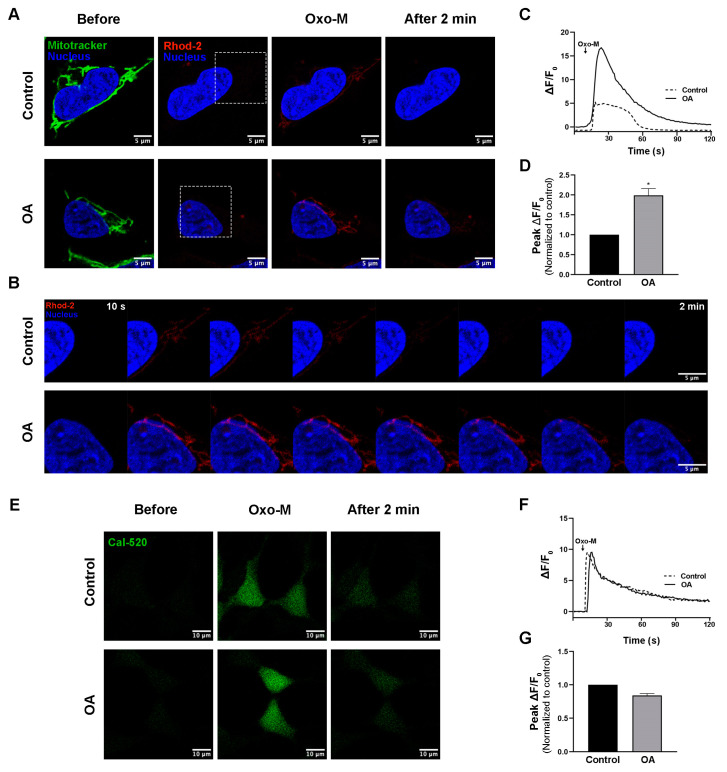
Effects of PP2A inhibition on mitochondrial Ca^2+^ in SH-SY5Y cells. (**A**) Representative time-lapse images of control and okadaic acid-treated cells loaded with Mitotracker and Rhod-2 fluorescence dye. The images were captured at 880 ms intervals and show the cells before, at peak intensity, and 2 min after Oxo-M stimulation. (**B**) Magnified images from the white boxes in (**A**) showing 10 s interval time lapses. (**C**) Plots of Rhod-2 fluorescence intensity from (**A**) are shown as ∆F/F_0_ vs. time (s). (**D**) Bar graph representing the peak value of ∆F/F_0_ normalized with the control. (**E**) Representative images of Cal-520 fluorescence in the cells before, at peak intensity, and 2 min after Oxo-M stimulation. (**F**) Plots of Cal-520 fluorescence intensity from (**E**) are shown as ∆F/F_0_ vs. time (s). (**G**) Bar graph representing the fluorescence intensity of the peak value of ∆F/F_0_ normalized with the control. Data represent means ± SEMs of four independent experiments for mitochondrial Ca^2+^ levels and of three independent experiments for cytosol Ca^2+^ levels. * *p* < 0.05 (Student’s *t*-test). Scale bars are 5 μm and 10 μm.

The imbalance of mitochondrial Ca^2+^ in neuronal cells results in oxidative stress and neurodegeneration. Since MAMs have a major role in Ca^2+^ transfers to mitochondria, we investigated whether PP2A inhibition also disrupts mitochondrial Ca^2+^ influx in SH-SY5Y cells. First, mitochondrial Ca^2+^ basal levels were measured using Rhod-2 fluorescence dye, an acetoxymethyl (AM) ester fluorescence dye that can penetrate cells and become trapped inside the mitochondrial matrix. After 10 h of okadaic acid treatment, Rhod-2-positive cells were detected using flow cytometry. The percentage of Rhod-2-positive cells was not different from that for the control (Figure S2), suggesting that the basal level of mitochondrial Ca^2+^ was not affected by okadaic acid treatment. Then, we monitored the effect of PP2A inhibition (okadaic acid treatment) on mitochondrial Ca^2+^ dynamics after transiently stimulating Ca^2+^ release from the ER using Oxo-M, an M3 muscarinic acetylcholine receptor agonist [26]. SH-SY5Y cells were incubated with Rhod-2 fluorescence dye and Mitotracker to label mitochondrial Ca^2+^ and mitochondrial structure, respectively. Following oxo-M application, the rates and amounts of Ca^2+^ entering mitochondria were greater in the cells treated with okadaic acid compared to the control (Figure 2A,B), implying that mitochondrial Ca^2+^ influx was elevated. The peak values (peak ΔF/F_0_) were higher in okadaic acid-treated cells compared to the control (Figure 2C,D). The enhanced mitochondrial Ca^2+^ influx upon stimulation of Ca^2+^ release from the ER suggests that PP2A inhibition induces a greater number of ER and mitochondria contacts than the baseline. This result is consistent with the increase in IP3R1/VDAC1 interaction (MAM) we observed, shown in Figure 1C,D. In addition, to test whether PP2A inhibition affects cytosolic Ca^2+^ dynamics in response to Oxo-M stimulation, cells in both conditions were incubated with a Cal-520 fluorescence dye before oxo-M stimulation. Neither the basal levels nor the peak ΔF/F_0_ of cytosol Ca^2+^ was different between the okadaic acid-treated and the control (Figure 2E–G) cells, implying that PP2A inhibition only disrupted mitochondrial Ca^2+^, which was likely due to an increase in the formation of MAMs.

### 3.3. PP2A Inhibition Decreased Mitochondrial Membrane Potential and Mitochondrial Fission 

**Figure 3:**
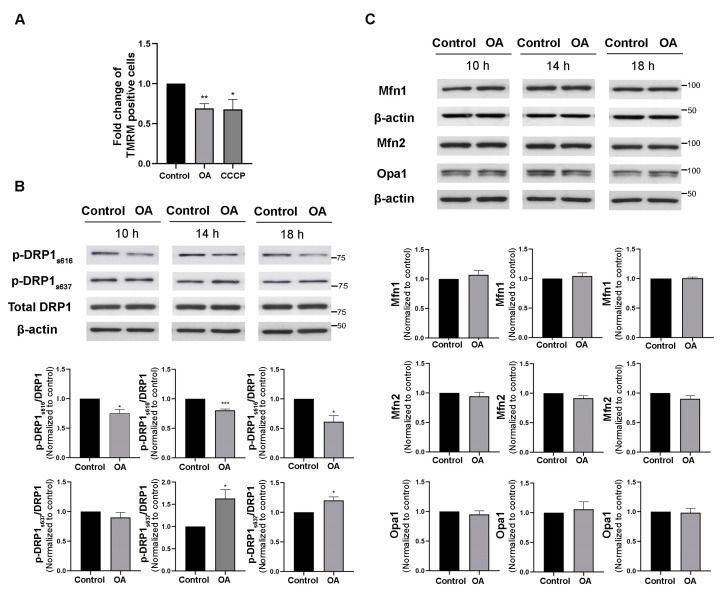
Effects of PP2A inhibition on mitochondrial membrane potential and dynamics. (**A**) Bar graph representing the fold change of TMRM-positive cells normalized with the control. Western blot analysis of (**B**) fission markers, including p-DRP_s616_, p-DRP_s637_, and DRP1, and (**C**) fusion markers, including mfn1, mfn2, and Opa-1, at 10 h, 14 h, and 18 h after okadaic acid treatment. The bar graphs show quantifications of the blots normalized with b-actin. Data represent means ± SEMs of three independent experiments. * *p* < 0.05, ** *p* < 0.01, *** *p* < 0.001 (Student’s *t*-test).

Healthy and functioning mitochondria establish and maintain specific membrane potentials, which can be observed by TMRM, a cell-permeant dye that accumulates in active mitochondria with intact membrane potentials. Mitochondrial Ca^2+^ overloading has been shown to induce the collapse of mitochondrial membrane potentials [22]. Therefore, we hypothesized that the increase in MAMs and mitochondrial Ca^2+^ observed after PP2A inhibition might also affect mitochondrial membrane potential. To test this hypothesis, SH-SY5Y cells were incubated with TMRM fluorescence dye for 45 min after being treated with 10 nM okadaic acid for 10 h. CCCP, an agent that disrupts mitochondrial membrane potential, was used as a positive control. TMRM fluorescence intensity in each group of cells was then detected by flow cytometry. We found that TMRM-positive cells were reduced in cell cultures treated with okadaic acid for 10 h (Figure 3A). The results indicate that PP2A inhibition caused loss of mitochondrial membrane potential. 

Mitochondrial dynamics, including fusion and fission, play a role in maintaining mitochondrial membrane potential. Several studies have shown that mitochondrial dynamics are closely related to ER-mitochondria contacts [14,27]. To investigate whether PP2A inhibition affects mitochondrial dynamics, mitochondrial fusion and fission protein markers were evaluated in okadaic acid-treated cells using Western blot analysis. Mitochondrial fission is promoted by phosphorylation of DRP1 at ser616 (p-DRP1_s616_). We found that p-DRP1_s616_ was significantly decreased after 10 h, 14 h, and 18 h of okadaic acid treatment (Figure 3B). In addition, phosphorylation of DRP1 at ser637 (p-DRP1_s637_), which inhibits mitochondrial fission, was significantly increased after 14 h and 18 h of okadaic acid treatment (Figure 3B). These results suggest that PP2A inhibition interferes with mitochondrial fission. In contrast, the mitochondrial fusion markers mfn1, mfn2, and opa1 were not altered at any timepoint of okadaic acid exposure, indicating that mitochondrial fusion was not affected by PP2A inhibition (Figure 3C). Altogether, these data provide evidence that PP2A inhibition interferes with mitochondrial dynamics, with specific effects on the fission process, without disturbing mitochondrial fusion.

## 4. Discussion

It has been proposed that MAM alteration is linked to Alzheimer’s disease, but the underlying mechanisms remain unclear. PP2A is also known to be decreased in AD brains, but the link between MAMs and PP2A in neuronal cells has never been investigated. In this study, we show that PP2A inhibition by okadaic acid promotes MAM formation, suggesting a reciprocal relationship between PP2A activity and MAM formation in SH-SY5Y cells, a neuron-like cell line. This is consistent with a previous study that showed that increased PP2A activity during high-glucose treatment reduced MAM formation in hepatocytes [22]. Based on this evidence, PP2A activity might be a critical modulator of MAM formation. Several possible mechanisms may underlie PP2A inhibition-driven effects on MAM formation, which should be further investigated. For example, PP2A inhibition might modify mitochondria surfaces or ER contents, leading to an increase in MAMs. Additionally, various proteins have been shown to be localized at the ER–mitochondria interface in a variety of rodent organs and brains, including IP3Rs, VDAC1, phosphatidylserine synthase-1 (PSS1), phosphofurin acidic cluster sorting protein-2 (PACS-2), and sigma 1 receptor (σ1R) [28,29]. Thus, PP2A inhibition might directly affect the levels of these proteins in MAM fractions. Furthermore, a recent study in brain endothelial cells suggests that PP2A inhibition drives the rearrangement of microtubules and the actin cytoskeleton [30]. Other studies have proposed the roles of glucose-regulated protein 75 (GRP75), a key chaperone protein expressed at the MAM interface, in the regulation of ER-mitochondrial Ca^2+^ transfer, while earlier studies showed that increased GRP75 affected mitochondrial function, leading to cell death [31]. In contrast, genetic ablation of GRP75 can weaken the ER-mitochondrial connection, which was shown to protect against mitochondrial dysfunction and cell death in a model of glutamate-induced oxidative stress [32]. Moreover, PP2A has also been shown to indirectly regulate heat shock protein expression [33]. Hence, it is possible that PP2A might affect the expression levels or function of GRP75, which in turn can alter the levels and function of MAMs.

MAMs facilitate Ca^2+^ transfer from ER to mitochondria [34,35,36]. Our data showed that mitochondrial Ca^2+^ influx was clearly increased in cells treated with PP2A inhibition, without changes in basal cytosolic Ca^2+^ levels. Therefore, increased mitochondrial Ca^2+^ dynamics may be facilitated by the upregulation of MAMs. A previous study in brain endothelial cells showed that okadaic acid can increase mitochondrial Ca^2+^ through the upregulation of sigma-1 receptors. The sigma-1 receptor, a molecular chaperone located at the ER–mitochondria interface on both the ER side and the mitochondrial side, is involved in calcium homeostasis through the stabilization of IP3R3 [30,37,38]. Another study also showed an increase in expression levels of this receptor in the brain of an AD mouse model prior to Aβ plaque formation [9]. It is possible that the increase in mitochondrial Ca^2+^ transfer observed in our model was concomitantly mediated by an increase in sigma-1 receptors and elevated levels of MAMs. Future studies are required to provide more insights into these mechanisms. 

MAMs have been shown to regulate mitochondrial membrane potential, metabolism, and dynamics [14,39]. Mitochondrial fission is an acute and adaptive response in injured neurons [40]. It helps to eliminate impaired mitochondria by segregating damaged parts of mitochondria that may further undergo mitophagy [41]. Here, we found that mitochondrial membrane potential was disrupted with PP2A inhibition, along with an increase in MAMs and a decrease in mitochondrial fission. Thus, the reduction in mitochondrial fission after PP2A inhibition may cause the accumulation of depolarized mitochondria, which may be mediated through an increase in MAMs. The reduced mitochondrial fission observed in our model might indicate that the neurons lack this adaptive response. When cells fail to eliminate damaged mitochondria, this can lead to high levels of stress and trigger excessive mitochondrial fission, thereby inducing neuronal dysfunction, as found in AD brains. However, it is unknown whether this decrease in mitochondrial fission during PP2A inhibition is a direct consequence of MAM alteration. PP2A inhibition may disrupt MAM formation, leading to the alteration of signaling pathways that interfere with mitochondrial fission. It is also possible that PP2A inhibition directly disrupts the activities of kinases that regulate mitochondrial dynamics. Further studies are required to prove these hypotheses.

Our study used SH-SY5Y cells as a model for investigating the mechanisms of MAM modulation during PP2A inhibition. Although this cell line has been used to examine MAM formation in several studies [7,8,10,42,43], it might not be able to represent what happens in primary neurons. Further studies of how PP2A inhibition affects MAM formation in neurons derived from either AD mouse models or human induced pluripotent stem cells from AD patients are required to recapitulate the full aspects of neuropathology. In addition, knocking down PP2A in these neurons will also provide further information on its roles in MAM formation and functions, as well as elucidate the signaling pathways involved.

Taken together, our results demonstrate that the reduction in PP2A activity, which mimics the condition found in early AD brains, is linked to alterations in MAM contacts and functions. This study highlights another pathological role of PP2A activity besides tau hyperphosphorylation in AD. In addition, several studies have proposed that increasing PP2A activity may be an effective therapy for patients with AD [44,45]. The effects of PP2A inhibition found in this study could support this hypothesis, apart from the well-studied effects of decreased PP2A activity on tau hyperphosphorylation and amyloid plaque formation. Further investigation is necessary to gain a better understanding of the mechanisms governing PP2A-mediated regulation of MAM formation and contacts, which might lead to new strategies designed to slow or reverse cellular dysfunctions that are mediated by MAMs.

## Data Availability

The data presented in this study are available on request from the corresponding author.

## Supplementary Materials

The following supporting information can be downloaded at: https://www.mdpi.com/article/10.3390/biomedicines11041011/s1, Figure S1: PLA negative controls; Figure S2: Basal levels of mitochondrial Ca^2+^ are not altered after okadaic acid exposure.
