# Supplementary material for: The Effects of PP2A Disruption on ER-Mitochondria Contact and Mitochondrial Functions in Neuronal-like Cells
Source: Biomedicines. 2023 Mar 26;11(4):1011. doi: 10.3390/biomedicines11041011 (PMC10135475; doi:10.3390/biomedicines11041011)
Supplement: Supplementary file 1 [file biomedicines-11-01011-s001.zip › biomedicines-2250259-supplementary.pdf]

Supplementary Materials

The effects of PP2A disruption on ER-mitochondria contact and mitochondrial functions in neuronal cells

Phaewa Chaiwijit , Kwanchanok Uppakara, Nithi Asavapanumas and Witchuda Saengsawang

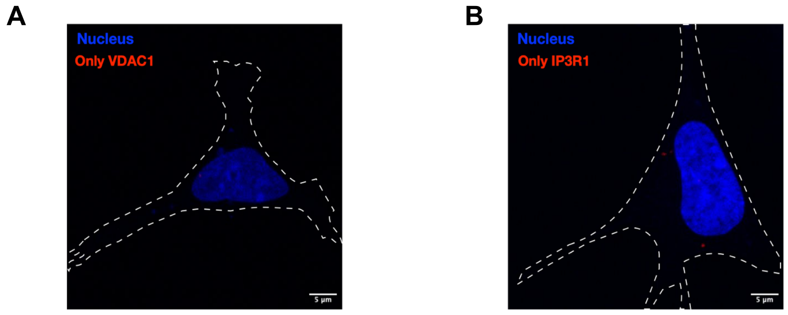

Figure S1 PLA negative controls. The PLA was performed in SHSY5Y cells as described in materials and methods without IP3R1 antibody **(A)** or without VDAC1 antibody **(B)**. Scale bars are 5 µm

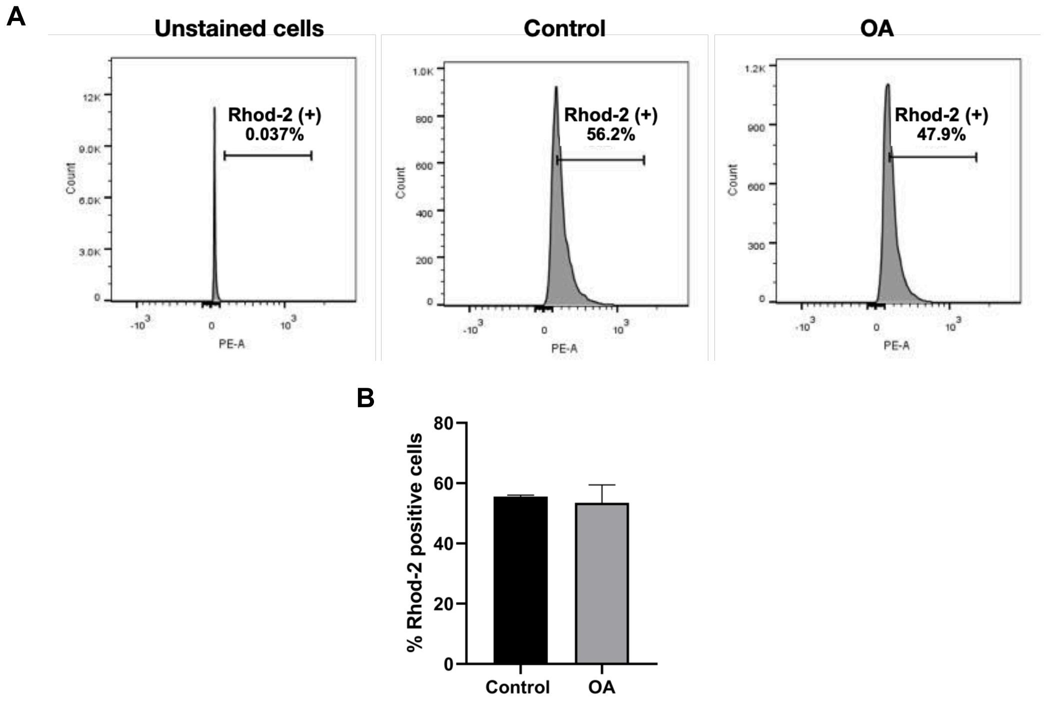

Figure S2 Basal level of mitochondrial Ca^{2+} is not altered after okadaic acid exposure. **(A)** Representative figures of percentages of rhod-2 positive cells. Unstained cells are used as negative control and quantification of gated cell are shown in bar chart of rhod-2 positive cells **(B).** Data represent mean ± S.E.M. of three independent experiments.

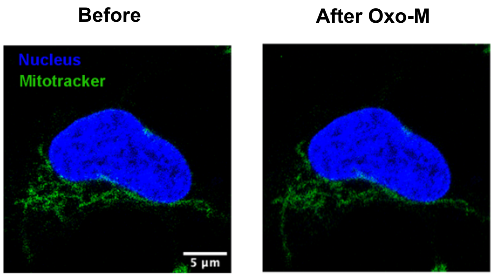

Figure S3 Representative time-lapse images of control cells loaded with Mitotracker fluorescence dye. The images were captured at 880 ms intervals and are shown as before and 2 min after Oxo-M stimulation.
